# Amino Acid Metabolism-Related lncRNA Signature Predicts the Prognosis of Breast Cancer

**DOI:** 10.3389/fgene.2022.880387

**Published:** 2022-05-13

**Authors:** Yin-wei Dai, Zhi-kai Wen, Zhi-xuan Wu, Hao-dong Wu, Lin-xi Lv, Cong-zhi Yan, Cong-hui Liu, Zi-qiong Wang, Chen Zheng

**Affiliations:** ^1^ Department of Breast Surgery, The First Affiliated Hospital of Wenzhou Medical University, Wenzhou, China; ^2^ Department of Hepatopancreatobiliary Surgery, The First Affiliated Hospital of Wenzhou Medical University, Wenzhou, China; ^3^ Wenzhou Medical University, Wenzhou, China

**Keywords:** amino acid metabolism, breast cancer, long non-coding RNA, prognostic signature, prognostic model, immunity

## Abstract

**Background and Purpose:** Breast cancer (BRCA) is the most frequent female malignancy and is potentially life threatening. The amino acid metabolism (AAM) has been shown to be strongly associated with the development and progression of human malignancies. In turn, long noncoding RNAs (lncRNAs) exert an important influence on the regulation of metabolism. Therefore, we attempted to build an AAM-related lncRNA prognostic model for BRCA and illustrate its immune characteristics and molecular mechanism.

**Experimental Design:** The RNA-seq data for BRCA from the TCGA-BRCA datasets were stochastically split into training and validation cohorts at a 3:1 ratio, to construct and validate the model, respectively. The amino acid metabolism-related genes were obtained from the Molecular Signature Database. A univariate Cox analysis, least absolute shrinkage and selection operator (LASSO) regression, and a multivariate Cox analysis were applied to create a predictive risk signature. Subsequently, the immune and molecular characteristics and the benefits of chemotherapeutic drugs in the high-risk and low-risk subgroups were examined.

**Results:** The prognostic model was developed based on the lncRNA group including LIPE-AS1, AC124067.4, LINC01655, AP005131.3, AC015802.3, USP30-AS1, SNHG26, and AL589765.4. Low-risk patients had a more favorable overall survival than did high-risk patients, in accordance with the results obtained for the validation cohort and the complete TCGA cohort. The elaborate results illustrated that a low-risk index was correlated with DNA-repair–associated pathways; a low *TP53* and *PIK3CA* mutation rate; high infiltration of CD4^+^ T cells, CD8^+^ T cells, and M1 macrophages; active immunity; and less-aggressive phenotypes. In contrast, a high-risk index was correlated with cancer and metastasis-related pathways; a high *PIK3CA* and *TP53* mutation rate; high infiltration of M0 macrophages, fibroblasts, and M2 macrophages; inhibition of the immune response; and more invasive phenotypes.

**Conclusion:** In conclusion, we attempted to shed light on the importance of AAM-associated lncRNAs in BRCA. The prognostic model built here might be acknowledged as an indispensable reference for predicting the outcome of patients with BRCA and help identify immune and molecular characteristics.

## Introduction

Breast cancer (BRCA) is the most frequent female malignancy and is potentially life threatening. Moreover, BRCA has one of the highest lethality rates among the female malignant tumors (Siegel et al., 2019; Siegel et al., 2020; Siegel et al., 2021). The BRCA incidence rates continue to increase by about 0.5% per year (Siegel et al., 2020; Siegel et al., 2021). Currently, dozens of treatments, including surgery, hormonal therapy, radiation therapy, and chemotherapy, are used to manage female BRCA. Nevertheless, most patients with BRCA are still at risk of having adverse outcomes, even for those who receive therapy in the early stage of the disease (Ciriello et al., 2015). In recent years, researchers have dedicated increased efforts to proving that the AAM is dramatically associated with BRCA development (Cha et al., 2018; Deyu Zhang et al., 2021; Morotti et al., 2021) and have demonstrated that BRCA is infiltrated by many types of immune cells and own a high level of immunogenicity, illustrating the hypothesis that immune cell infiltration plays an indispensable role in the clinical prognosis of BRCA (Lehmann et al., 2011; Loi et al., 2014; Denkert et al., 2018; Pruneri et al., 2018). Moreover, the tumor immune microenvironment participates significantly in the development of BRCA. Tumor-infiltrating lymphocytes have been shown to be correlated with the outcome of this disease (Pruneri et al., 2018).

Tumor-infiltrating lymphocytes have been shown to be correlated with the outcome of this disease (Adams et al., 2014; Ali et al., 2016; Stanton and Disis, 2016; Lee et al., 2018).

Altered metabolism is a hallmark of cancer, and the reprogramming of the energy metabolism has historically been acknowledged as a general phenomenon underlying tumors (Pei-Hsuan Chen et al., 2019; Faubert et al., 2020; Wang et al., 2018). One of the best-known alternative theories on cancer development is the “Warburg effect,” which consists of the continued activation of aerobic glycolysis in cancer cells (Hanahan and Weinberg, 2011). Furthermore, the AAM has been shown to be strongly correlated with the evolution and progression of human malignancies. Glutamine, serine, and glycine, for instance, are vital nutrients for tumor growth and maintenance. Moreover, myc overexpression influences the cellular glutamine levels by activating the transcription of GLS1 and the glutamine transporter SLC1A5 (Gao et al., 2009). In turn, phosphoglycerate dehydrogenase (PHGDH), which is a crucial enzyme in the serine synthesis pathway, is greatly upregulated in breast cancer cells (Dias et al., 2019). Similarly, immune cells require amino acids as a vital source of nutrition. The AAM can regulate immune effector protein activity (Kelly and Pearce, 2020). For example, activated CD8^+^ T cells exhibit higher levels of Slc7a5 and Slc1a5 on the cell surface compared with naïve CD8^+^ T cells. Activated T cells need a considerable amount of amino acids to maintain growth by improving transporter expression (Kelly and Pearce, 2020). Recent studies have demonstrated that the combination of a glutamine antagonist with anti-PD-1 therapy had a more obvious anti-tumor effect than did the anti-PD-1 therapy alone, and did not cause immune cell failure (Leone et al., 2019). In addition, the combination of the ladiratuzumab vedotin LIV-1 directed which is a transmembrane protein with zinc transporter and metalloproteinase activity (Nagayama et al., 2020) and anti-PD-1 therapy have been highlighted in breast cancer, particularly Triple negative breast cancer (TNBC) (Rizzo et al., 2022). There are many TNBC patients who benefit from immune checkpoint blockade therapy (Rizzo et al., 2021; Rizzo and Ricci, 2021).

LncRNAs, which are RNA molecules with a length of about 200 nt, can adjust the expression of genes (Tao Zhang et al., 2021). With the exception of gene regulation, lncRNAs participate in numerous biological regulatory processes, including those involved in the appearance, development, and metastasis of tumors (Gupta et al., 2010). Moreover, lncRNAs have a great impact on the regulation of metabolism (Guo et al., 2021; Tan et al., 2021). lncRNAs can directly modulate the posttranslational modification of key metabolic enzymes, lncRNAs can also indirectly regulate metabolic pathways through posttranslational modifications (Tan et al., 2021). It has been proved by experiments that the lncRNA XLOC_006390 stablized c-Myc by preventing its ubiquitination, increasing the expression of glutamate dehydrogenase 1 (GDH1), and subsequently stimulating the production of alpha-ketoglutarate (α-KG). Excess α-KG supplied the tricarboxylic acid (TCA) cycle and facilitated glutamate metabolism, promoting pancreatic cancer growth (He et al., 2020). Meanwhile, LncRNA EPB41L4A-AS1 regulates glycolysis and glutaminolysis by mediating nucleolar translocation of HDAC2 (Liao et al., 2019). However, to date, AAM-related lncRNAs have not been used to predict overall survival (OS) in patients with BRCA. Whether amino acid metabolization-related lncRNAs participate in the immune regulation of BRCA remains ambiguous.

In this study, we used the TCGA BRCA database, from which genomic and transcriptome data (RNA-seq) are accessible. In *cross-*validation analyses, the dataset was stochastically divided into training and *validation* groups. Finally, nine lncRNAs that were correlated with BRCA outcomes were identified. According to the expression levels of these nine lncRNAs, a risk score prognostic model for BRCA was created according to the patients in the training cohort, and the vital prognostic values of this model were further acknowledged in the patients in the validation cohort and the patients in the whole cohort. The relationships between the risk score subtypes and immune checkpoints, the proportions of 22 immune cells [B cells naive, B cells memory, plasma cells, T cells CD8, T cells CD4 naive, T cells CD4 memory resting, T cells CD4 memory activated, T cells follicular helper, T cells gamma delta, T cells regulatory (Tregs), NK cells resting, NK cells activated, monocytes, macrophages M0, macrophages M1, macrophages M2, dendritic cells resting, dendritic cells activated, mast cells resting, mast cells activated, eosinophils, and neutrophils], and intrinsic molecular subtypes were also illustrated (Newman et al., 2015; Becht et al., 2016). Lastly, the difference between the two sets regarding sensitivity to chemotherapy was predicted.

## Methods

### Data Source and Preprocessing

To collect the messenger RNA (mRNA) expression profiles and clinical information of patients with BRCA, comprehensive computerized searches of TCGA datasets (https://portal.gdc.cancer.gov/repository) were conducted. Samples with a follow-up time of less than 1 month or male BRCA samples were excluded. A total of 1208 TCGA female patients with BRCA for whom lncRNA expression profiles were available were analyzed in the present study. The AAM-related gene sets (REACTOME_METABOLISM_OF_AMINO_ACIDS_AND_DERIVATIVES) were derived from the Molecular Signatures Database v5.1 (MSigDB) (http://www.broad.mit.edu/gsea/msigdb/), which incorporated 374 genes in total. After assessing the overlap with genes in the TCGA RNA-seq datasets, 374 genes associated with AAM remained in study. The AAM-related lncRNAs were selected based on the criteria of *p* < 0.001 and Pearson’s correlation coefficient |> 0.4, as assessed using the limma R package (Schober et al., 2018). Moreover, the “limma” R package was used to identify the differentially expressed genes (DEGs), including lncRNAs, protein-coding genes, miRNAs, etc., between nontumor and tumor tissues, with a false discovery rate (FDR) < 0.05 and |log2FC| ≥ 1.

Here, we first explored the function of both downregulated and upregulated AAM-related DEGs. Subsequently, we applied a gene ontology (GO) analysis to assess the biological pathways related to the DEGs. A further functional analysis of biological processes (BPs), molecular functions (MFs) and cellular components (CCs) adjusted to the individually expressed AAM-related DEGs was performed based on Kyoto Encyclopedia of Genes and Genomes (KEGG) data using the R software, ggplot2 package.

### Development and Validation of the Prognosis Model

The 1,005 patients who had a survival time ≥30 days and complete follow-up information were stochastically split into the training and validation cohorts at a 3:1 ratio, for the building and validation of the accuracy of the prognostic model. A univariate Cox regression analysis was run to screen prognostic lncRNAs that were correlated (*p* < 0.05) with the overall survival (OS) of the patients in the training cohort. All 17 of these lncRNAs were further enrolled into a LASSO analysis for dimension reduction in the “glmnet” R package. Subsequently, a multivariate Cox analysis further selected nine lncRNAs according to the lowest Akaike information criterion value obtained for the 17 AAM-related lncRNAs with prognostic significance described above. The risk score of each patient derived from this prognostic signature was calculated based on the normalized expression level of AAM-related lncRNAs and corresponding regression coefficients. The computational formula used here was as follows:

$${\text{Risk Score}=\text{e}}^{\text{sum}\left(\text{each lncRNA's expression}\times \text{corresponding regression coefficient}\right)}$$

The patients in the training cohort were split into high-risk and low-risk sets based on the comparison of the median value of the risk score and the OS between the different groups using a Kaplan–Meier (K–M) analysis with a log-rank test. Subsequently, a time-dependent receiver operating curve (ROC) curve analysis was performed using the “survivalROC” R package, to assess the predictive veracity of the prognosis model. Furthermore, we applied univariate and multivariate Cox regression analyses to verify if the nine AAM-related lncRNAs were independent prognostic factors for BRCA. For the purpose of validating this model, the same regression coefficients, formula, and genes were applied in the analysis of the validation cohort and the complete cohort, to calculate the risk score.

### Comparison of the AAM-Related lncRNA Signature With Other BRCA Prognostic Models

To examine whether the model constructed here is more referential than are other BRCA prognostic models, we utilized a ROC for comparison with an 11-lncRNA signature (Shen et al., 2020), another 11-lncRNA [11 (2)-lncRNA] signature (Xiaoying Li et al., 2021), and an eight-lncRNA signature (Zhu et al., 2021). We obtained the correlative lncRNAs in these models from the literature, and the 1-, 3-, and 5-year OS ROC curves for the complete TCGA cohort were created. Finally, these lncRNA-based prognostic models were compared to illustrate the merits and shortcomings of each of them.

### Building of an lncRNA–mRNA Co-expression Network

For the purpose of demonstrating the interaction between the nine-lncRNA signature and their interrelated mRNAs, we applied the Cytoscape software (version 3.8.2,http://www.cytoscape.org/) to design and visualize an mRNA–lncRNA co-expression network.

### Gene Set Enrichment Analysis

We ran a gene set enrichment analysis (GSEA) 4.2.1 (https://www.gsea-msigdb.org/gsea/msigdb) (Subramanian et al., 2005) to distinguish various functional phenotypes between the high-risk and low-risk sets. The mRNA expression profiles of BRCA samples in the TCGA datasets, which were separated into two groups based on risk score, were applied to KEGG gene sets. The study included enriched gene sets with *p* < 0.05, and 1,000 random sample permutations were acknowledged as being statistically significant. Their default values were used for the other parameters.

### Establishment of Predictive Nomograms

A nomogram was created on the ground that the results of the multivariate analysis in the R software package using the nomogram function from the “rms” library, to predict the 1-, 3-, and 5-year survival of patients with BRCA. Harrell’s concordance index (C-index) and calibration curve illustrate the predicting value of the nomograms and their discrimination performance.

### Immune-Related Features

The MCPcounter (Wang et al., 2019), ESTIMATE (Yoshihara et al., 2013), CIBERSORT (Newman et al., 2015; Charoentong et al., 2017), and single-sample gene set enrichment analysis (ssGSEA) (Yi et al., 2020) algorithms were compared to estimate the differences in cell immune responses or cellular components between the low-risk and high-risk groups. We used a heatmap and boxplots to illustrate differences in the immune response using the various algorithms. Furthermore, for the purpose of quantifying the tumor-infiltrating immune cell subgroups in the BRCA tumor microenvironment (TME) among the two groups, as well as for estimating their immune function, we used ssGSEA. We retrieved dozens of potential immune checkpoints from the literature.

### Drug Susceptibility and Mutation Analysis

For the purpose of determining the somatic mutations of patients with BRCA in the high- and low-risk sets, the mutation annotation format from the TCGA database was created utilizing the “maftools” R package. The tumor mutation burden (TMB), which is described as mutations/megabase (mutations/Mb), is an effective biomarker for predicting the efficacy of immunotherapy. The TMB score for each sample with BRCA in the two groups was calculated. For the purpose of exploring the differences in the responses to chemotherapeutic drugs between the two groups, we analyzed the semi-inhibitory concentration (IC_50_) values of the chemotherapeutic drugs that are usually employed to treat BRCA using the “pRRophetic” package.

### Statistical Analysis

We applied R version 4.1.0. to run all statistical analyses. Significance was set at *p* < 0.05.

## Results

### Data Processing and Clinicopathological Features

**FIGURE 1:**
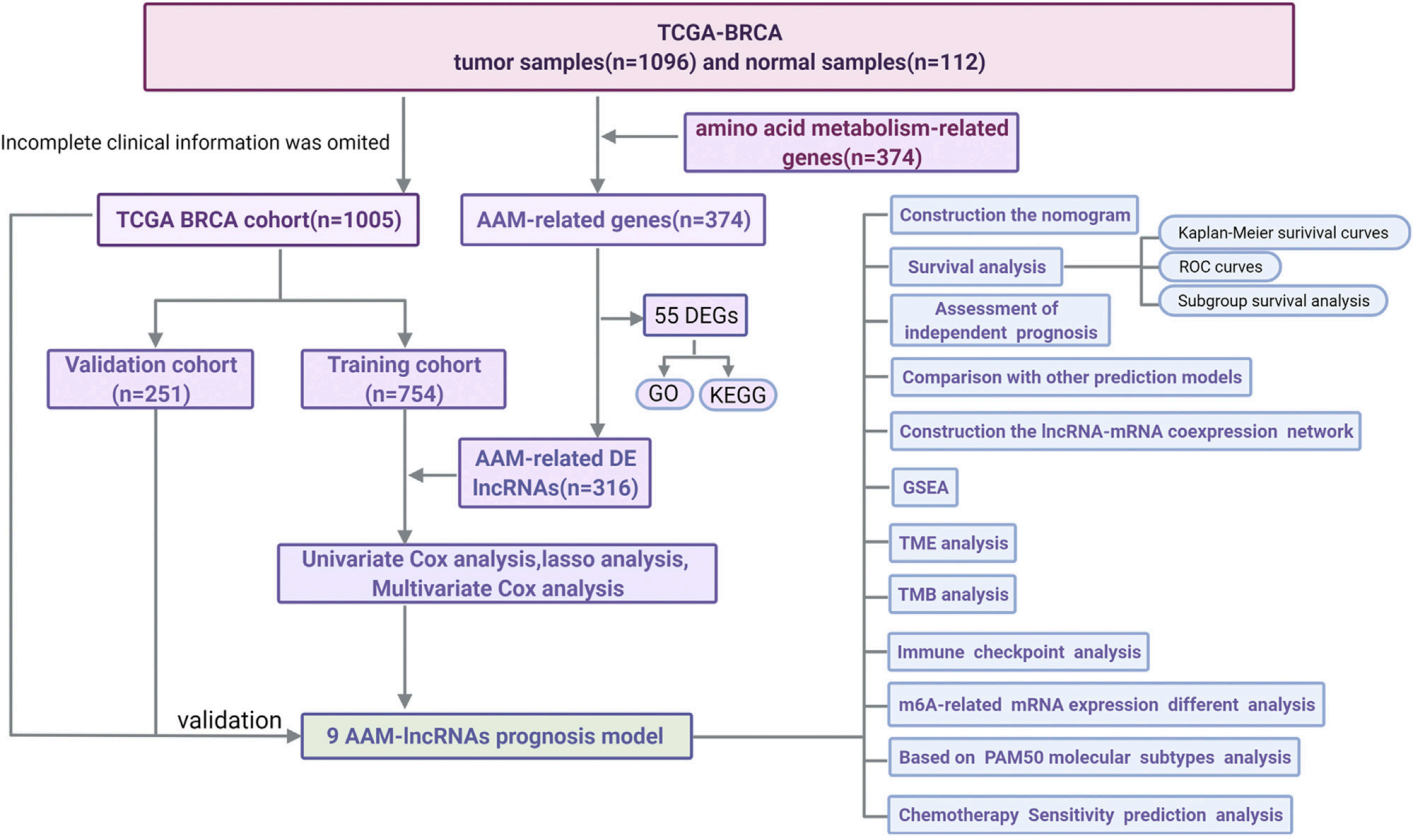
A flow chart of the data analysis and process.

A flow chart of the data analysis and process used in this study was drawn (Figure 1). After data partitioning and preprocessing, 765 patients with BRCA were distributed into the training set and 255 patients with BRCA were distributed into the validation set. Their clinicopathological features were outlined (Table 1).

**TABLE 1:** Clinicopathological features of the training set and the validation set.

Characteristic	TCGA set	Train set	Validation set	*p* value
Total cases	1,005	754	251	—
Age (years)	—	—	—	0.63
>65	278 (27.6%)	210 (27.9%)	68 (27%)	—
≤65	727 (72.3%)	544 (72.1%)	183 (72.9%)	—
Stage	—	—	—	0.21
I	186 (18.5%)	136 (18.0%)	50 (19.9%)	—
II	582 (57.9%)	433 (57.4%)	149 (59.3%)	—
III	222 (22.1%)	172 (22.8%)	50 (19.9%)	—
IV	15 (1.5%)	13 (1.7%)	2 (0.8%)	—
Fustat	—	—	—	0.058
Alive	892 (88.6%)	661 (87.7%)	231 (92.0%)	—
Dead	112 (11.4%)	93 (12.3%)	20 (8.0%)	—
Futime	—	—	—	0.3
>3 years	405 (40.3%)	315 (41.8%)	90 (35.9%)	—
≤3 years	600 (59.7%)	439 (58.2%)	161 (64.1%)	—

### Enrichment Analysis of AAM-Related Genes

**FIGURE 2:**
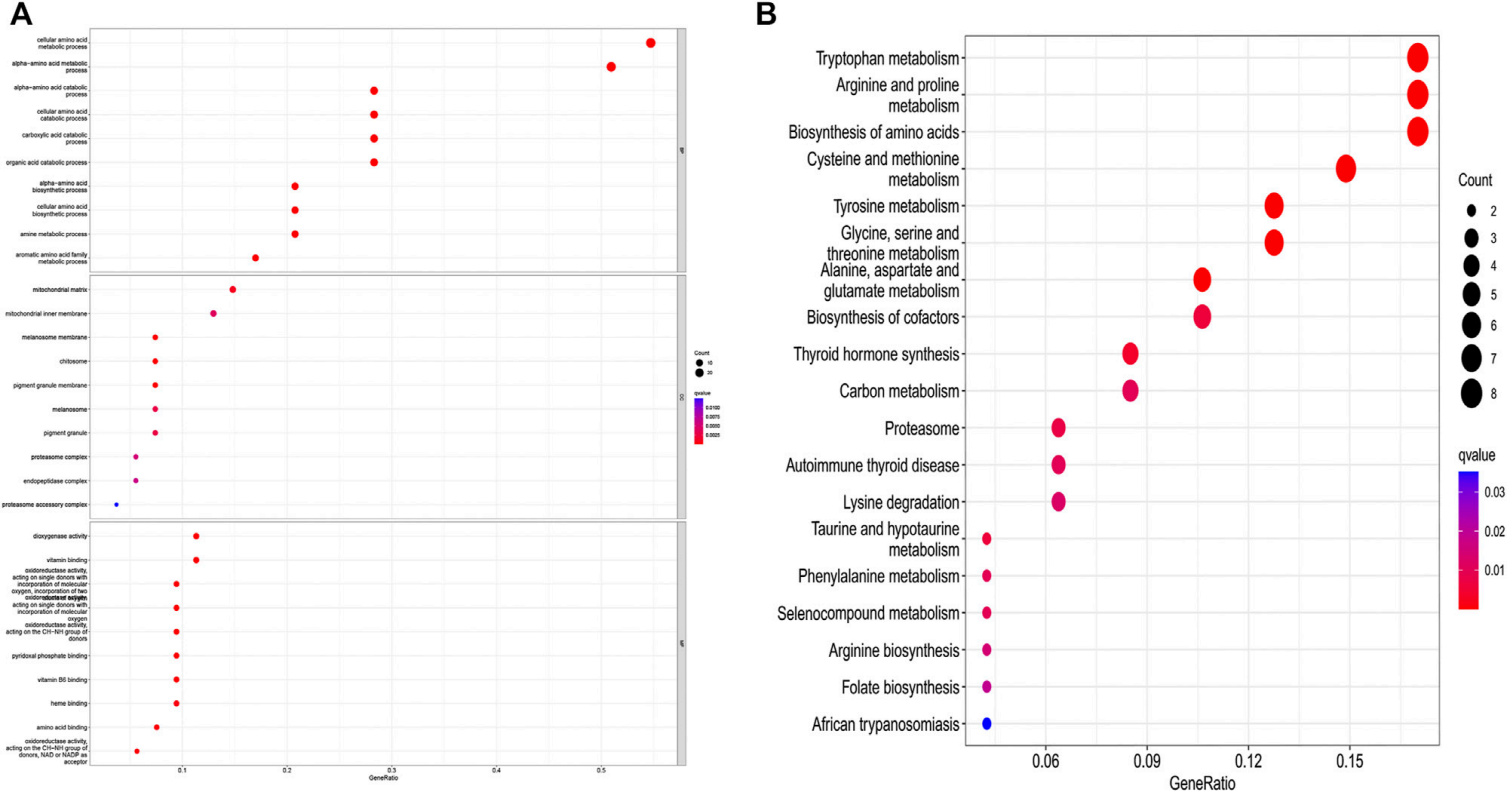
GO and KEGG analysis of amino acid metabolism-associated DEGs. **(A)** GO and **(B)** KEGG.

The differential gene expression analysis (1,096 tumors vs. 112 normal samples) identified 55 DEGs, 18 of which were downregulated and 37 of which were upregulated in the tumor samples compared with the normal samples (Supplementary Table S1). The DEGs in the BP category were involved in the production of alpha-amino acid and cellular amino acid metabolic processes, among others; the DEGs in the MF category mainly participated in the regulation of the production of dioxygenase and the binding of vitamins; the DEGs in the CC category were primarily upregulated in the mitochondrial matrix and mitochondrial inner membrane pathways. The KEGG-based analysis revealed that the DEGs primarily participated in arginine and tryptophan metabolism, the biosynthesis of amino acids, proline metabolism, cysteine and methionine metabolism, tyrosine metabolism, etc. (Figures 2A,B).

### AAM-Related lncRNA-Based Prognostic Signature

We identified 316 AAM-based lncRNAs (Supplementary Table S2). The univariate Cox analysis selected 17 AAM-associated lncRNAs (Figure 2A) that were included in the multivariate Cox analysis. Ultimately, nine lncRNAs (LIPE-AS1, AC124067.4, LINC01655, AP005131.3, AC015802.3, USP30-AS1, SNHG26, and AL589765.4) were selected as independent prognosis predictors of BRCA. Thus, we constructed a prognostic index for training cohort cancer samples using the following formula: risk score = (−0.606072 × expression of LIPE-AS1) + (−0.28451 × expression of AC124067.4) + (0.6666797 × expression of LINC01655) + (−0.988819 × expression of AP005131.3) + (−0.140664 × expression of AC008115.3) + (−0.767441 × expression of AC015802.3) + (−0.277495 × expression of USP30-AS1) + (−0.765508 × expression of SNHG26) + (0.138989 × expression of AL589765.4) (Table 2).

**TABLE 2:** Amino acid metabolism-related lncRNA-based prognostic signature.

Id	Coef	HR	HR.95L	HR.95H	*p* value
LIPE-AS1	−0.606072	0.5454891	0.2695349	1.10397	0.091994
AC124067.4	−0.28451	0.7523826	0.5595592	1.0116527	0.0596629
LINC01655	0.6666797	1.9477593	0.9994916	3.7956963	0.0501749
AP005131.3*	−0.988819	0.3720158	0.1479188	0.9356202	0.0356073
AC008115.3	−0.140664	0.8687813	0.7484818	1.0084158	0.0643486
AC015802.3	−0.767441	0.4641993	0.1671622	1.2890539	0.1408279
USP30-AS1*	−0.277495	0.7576792	0.5893708	0.9740519	0.0303807
SNHG26*	−0.765508	0.4650974	0.2367205	0.9138019	0.026313
AL589765.4***	0.138989	1.1491115	1.0659176	1.2387986	0.0002892

*p < 0.05,**p < 0.01,***p < 0.001.

### Survival Results and Multivariate Analysis

**FIGURE 3:**
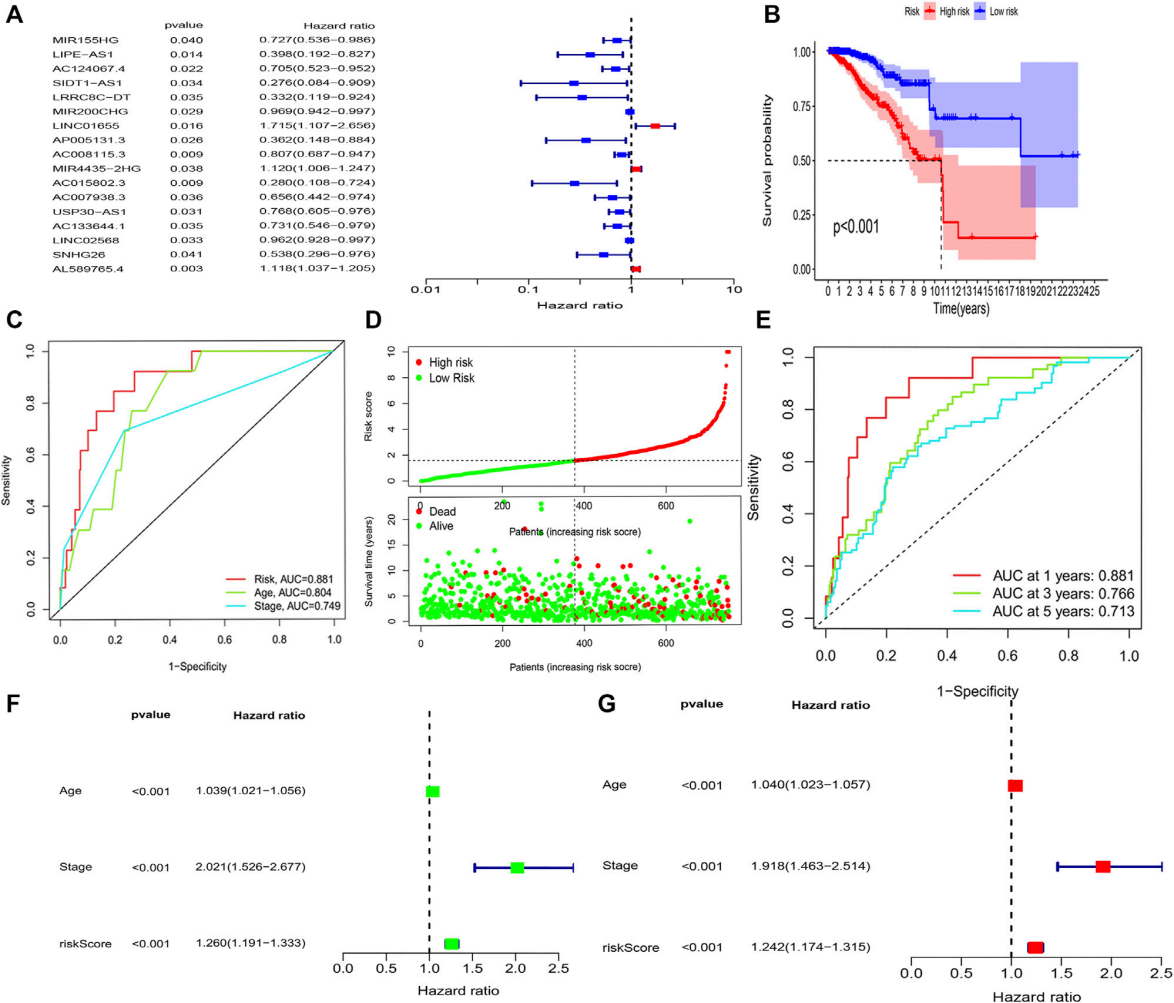
Amino acid metabolism-associated lncRNA signature based on training sets. **(A)** Univariate cox analysis **(B)** Kaplan–Meier curves, **(C)** multi-index ROC analysis, **(D)** risk score, and **(E)** time-dependent ROC analysis. Univariate and multivariate Cox analyses of the expression of AAM-related lncRNAs. **(F)** Univariate and **(G)** multivariate analyses.

The K-M survival analysis also showed that patients with BRCA in the high-risk group exhibited a lower OS (*p* < 0.001, Figure 3B). Concomitantly, The AUC of the signature lncRNAs was 0.881, illustrating a better predictive effect than that of the traditional clinicopathological features (Figure 3C). Interestingly, our heatmap showed that most of the novel lncRNAs exhibited a negative association with our risk model; additional experiments are necessary to address this issue (Figure 3D). The predictive value of the AUC of the novel lncRNA signature regarding the 1-, 3-, and 5-year survival rate was 0.881, 0.766, and 0.713, respectively (Figure 3E).

The hazard ratio and 95% CI of the risk score were 1.260 and 1.191–1.333 in the univariate Cox regression analysis (*p* < 0.001) and 1.242 and 1.174–1.315 in the multivariate Cox regression analysis (*p* < 0.001), respectively. These findings indicated that the nine-lncRNA signature was an independent prognosis factor of OS in patients with BRCA (Figures 3F,G).

**FIGURE 4:**
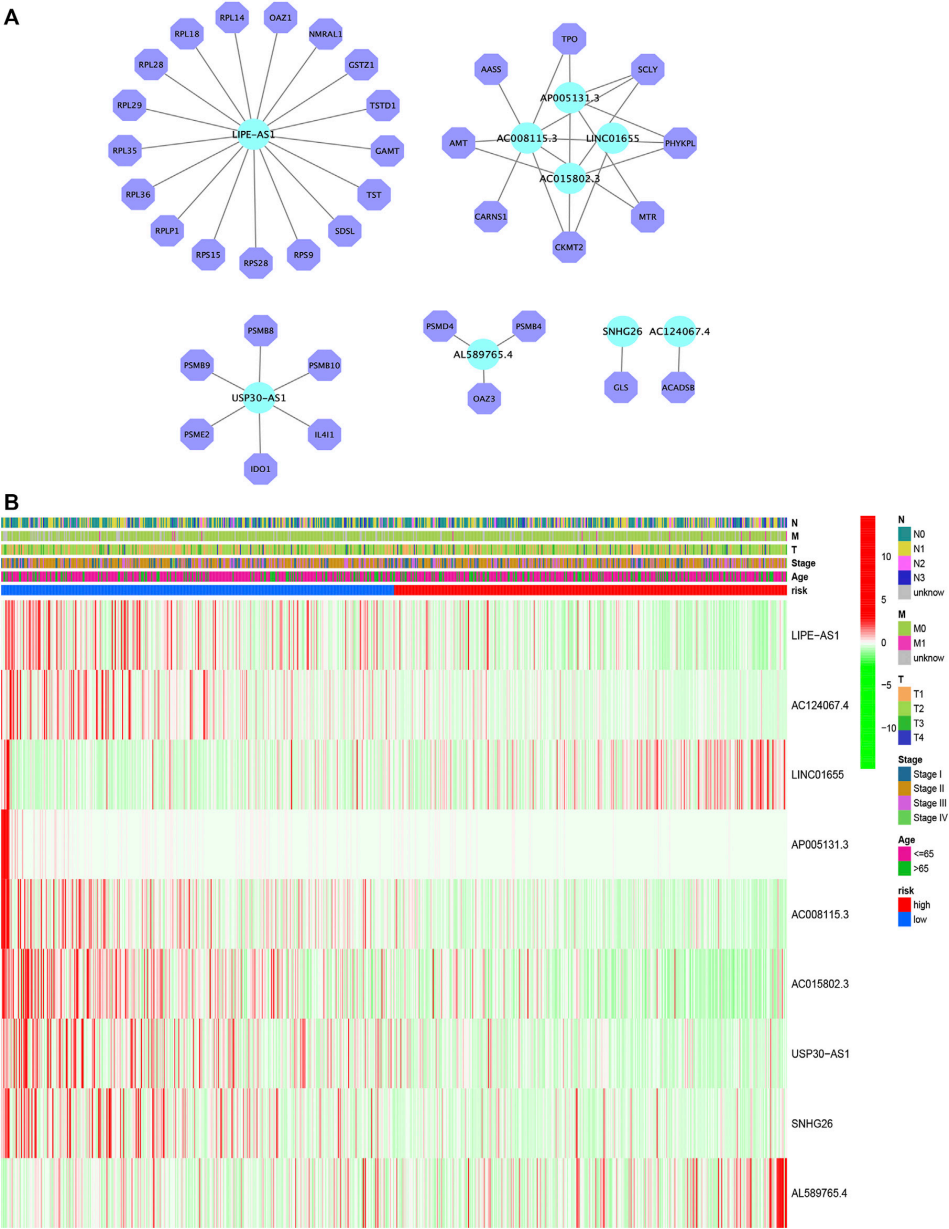
Construction of the mRNA–lncRNA regulatory network **(A)**. Heatmap of the clinicopathological manifestations and AAM-related lncRNA prognostic signature **(B)**.

**FIGURE 5:**
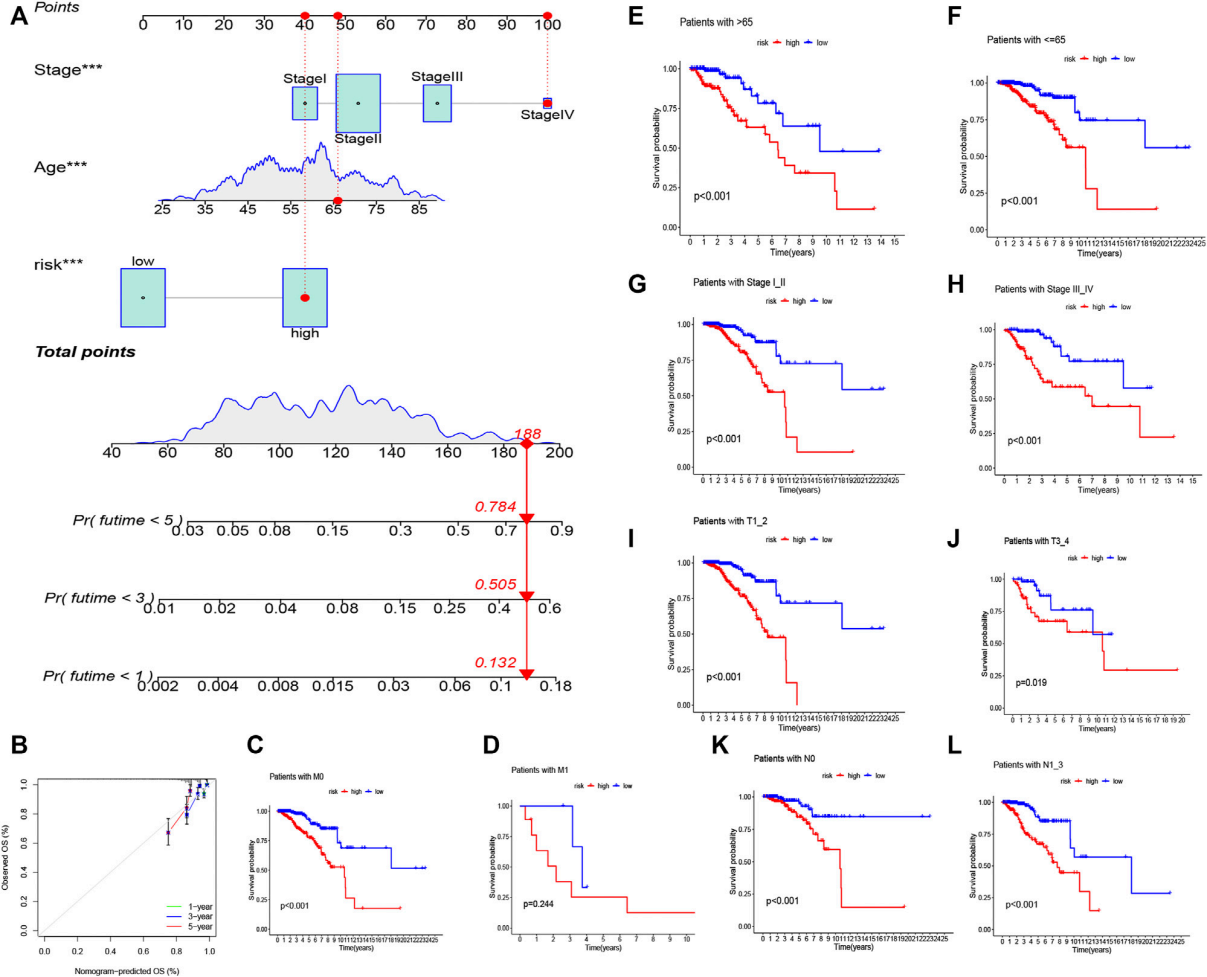
Nomogram of both prognostic AAM-associated lncRNAs and clinical–pathological factors **(A)**. Calibration plot for the nomogram **(B)**. Stratification analysis of the risk score in BRCA. **(C,D)** Age (age >65 and age ≤65 years). **(E,F)** Tumor stage (I–II or III–IV). **(G,H)** Tumor T stage (T1–2 or T3–4). **(I,J)** Tumor M stage (M0 or M1). **(K,L)** Tumor N stage (N0 or N1–3).

Figure 4A depicts the correlation between mRNAs and lncRNAs. The heatmap of the relationship between the clinicopathological manifestations and the AAM-related lncRNA prognostic index is also presented (Figure 4B). The hybrid nomogram (c-index = 0.754) encompassing the novel AAM-related lncRNA prognostic index and the clinicopathological characteristics is shown in Figure 5A. The calibration curve analysis revealed that the practical and the predicted 1-, 3-, and 5-year survival rates agreed with the reference curve (Figure 5B). These results suggested that the nomogram was accurate and stable; thus, it is suitable for implementation in the clinical management of patients with BRCA.

We ran a similar survival analysis by regulating the risk model using different physiological and clinical factors (e.g., age and tumor TNM stage). The K–M curves illustrated that the low-risk set had a better OS than the high-risk group in all subsets (Figures 5C–L). The K–M survival curves of the M1-stage subgroup were not statistically significant (*p* = 0.244). We considered that the fact that the number of patients was significantly low (only 20 samples) contributed to this observation; however, in general, the high-risk set had a worse OS than the low-risk set.

To confirm the prognostic accuracy of the risk score, the risk score each patient in the validation set and in the entire TCGA set was calculated and then split into two sets according to the median value. A survival analysis revealed a more favorable outcome in the low-risk set compared with the high-risk set (log-rank test; *p* < 0.001; Supplementary Figures S1A,B). An analysis of the 1-, 3-, and 5-year prognostic prediction classification efficiencies suggested that the risk score still had comparably high AUC values (Supplementary Figures S1C,D), suggesting that the risk model had an outstanding ability to predict the outcome of BRCA.

### Comparison of the AAM-Related lncRNA Signature With Other BRCA Prognostic Models

To determine if our nine-lncRNA signature is more accurate than other BRCA prognostic models, we compared it with an 11-lncRNA signature (Shen et al., 2020), another 11-lncRNA (11 (2)-lncRNA) signature (Xiaoying Li et al., 2021), and an eight-lncRNA signature (Zhu et al., 2021) for the entire TCGA cohort. However, the predictive accuracy of the AAM-related nine-lncRNA signature was greater than that of the remaining three prognostic models (Supplementary Figures S2A–H).

### Molecular Characteristics of the Different Risk Subgroups

**FIGURE 6:**
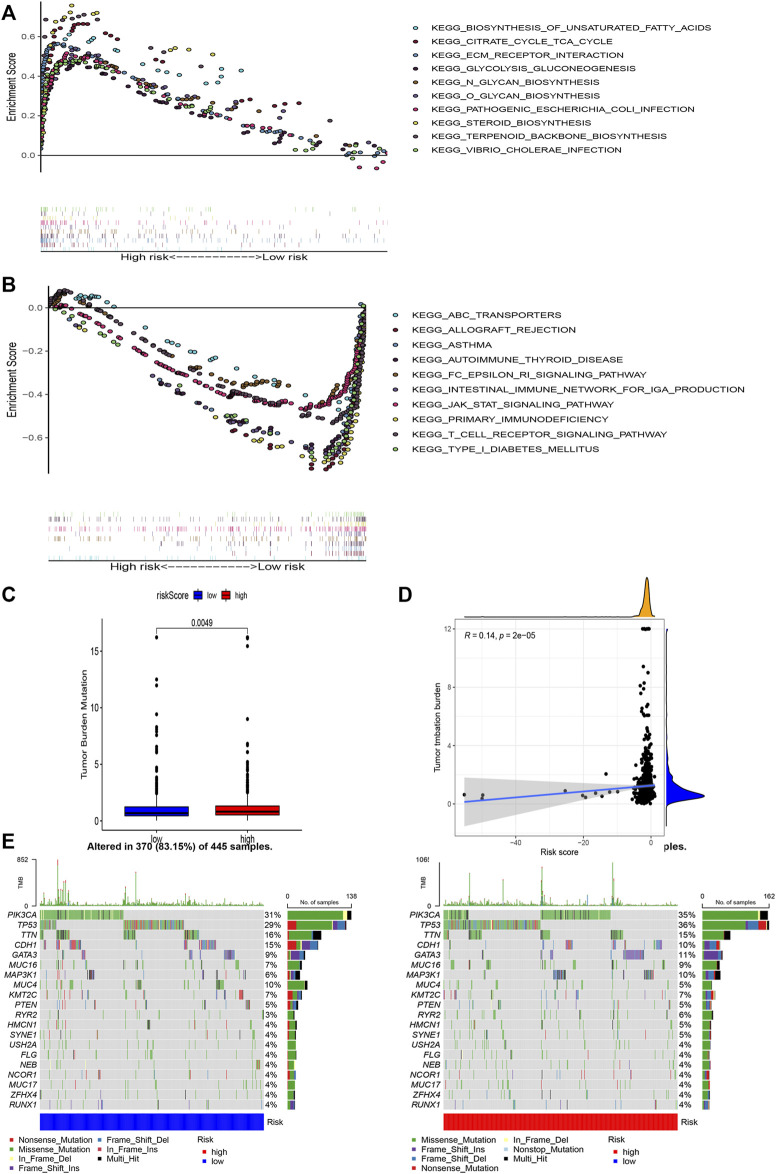
Gene enrichment analysis for AAM-related lncRNAs based on TCGA in the high **(A)** and low **(B)** BRCA risk groups. Correlation between the TMB and the two risk subsets **(C)**. Association between the TMB and risk score **(D)**. Prominently mutated genes in the patients with BRCA in the different risk subgroups. The mutated genes (rows, top 20) are ranked according to mutation rate; samples (columns) are arranged to emphasize the mutual exclusivity among mutations. The right panels depicts the mutation percentage, and the top panel indicates the overall number of mutations. The color coding indicates the mutation type **(E)**.

GSEA was used to perform functional annotations in the two groups. The gene sets of the low-risk samples were enriched in immune-related pathways (Figure 6B), whereas the gene sets of the high-risk samples were enriched in lipid and glucose metabolism pathways (Figure 6A; *p* < 0.05).

Next, we found that the risk score was slightly correlated with the TMB (*r* = 0.14, *p* < 0.001), as shown in Figures 6C,D.

Moreover, to shed light on the immunologic nature of the risk subgroups, gene mutations were explored in the different risk subgroups was explored. We noticed an apparently more frequent mutation in the high-risk subgroup vs. the low-risk subgroup (*p* = 0.0049, t-test) (Figure 6E). Moreover, the most frequent mutation type was missense mutation, followed by frameshift deletion and nonsense mutation. The top 20 genes with the highest mutation rates in the subgroups are illustrated in Figure 6E. The mutation rates of the TP53, PIK3CA, TTN, and CDH1 genes were higher than 10% in both groups. Mutation of the MAPK3K1 gene was more prominent in the high-risk subgroup, whereas mutation of the MUC4 gene was more prominent in the low-risk subgroup.

### Immune Characteristics of the Different Risk Subgroups

**FIGURE 7:**
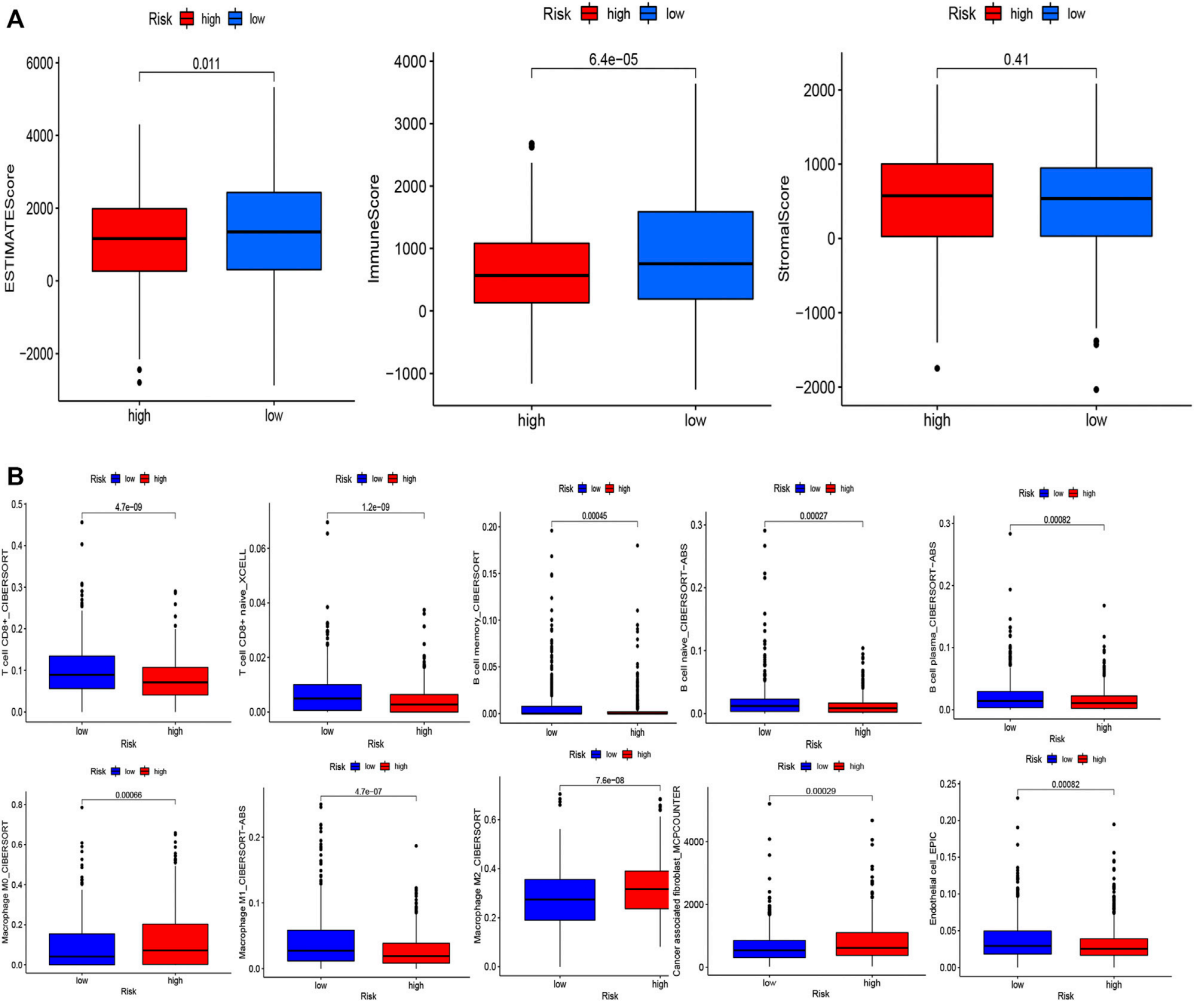
Evaluation of the TME and levels of lymphocyte infiltration in the two groups. **(A)** Associations between the risk score and the immune and stromal scores. **(B)** Associations between the risk score and immune cell types.

**FIGURE 8:**
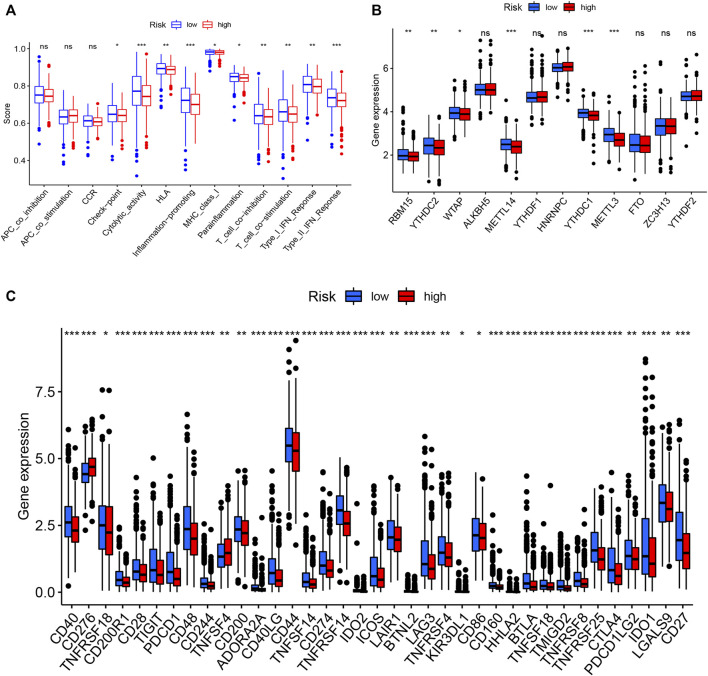
Immune cell infiltration levels and corresponding function determined by ssGSEA **(A)**. Expression of m6A-related genes in both groups **(B)**. Expression of immune checkpoint-related genes in both groups **(C)**.

To examine the composition of immune cells in the different risk subgroups, we used the Wilcoxon test to compare the distribution of immune cells among the different risk subgroups. We found that CD8^+^ T cells, plasma cells, naïve CD8^+^ T cells, M1 macrophages, memory B cells, and endothelial cells were more abundant in the low-risk subgroup, whereas M2 macrophages, M0 macrophages, and fibroblasts were more abundant in the high-risk subgroup (Figure 7B, Supplementary Figure S3). A low-Risk score was also deeply correlated with a high immune score (Figure 7A). We also investigated whether the prognostic value of risk scores stemmed from better immune control or from less-aggressive cancer growth. As shown in Figure 8A, we found that patients with a higher score on HLA, checkpoint, inflammation-promoting, parainflammation, T-cell co-inhibition, T-cell co-stimulation, type II IFN response, cytolytic activity, MHC-class-I, and type I IFN response had a better outcome. Therefore, we suggest that the prognostic value of the risk scores might result from both better immune control and less-aggressive cancer growth. The difference in the expression of immune checkpoints between the two subsets was further explored. The results of this analysis suggest a more abundant expression of PDCD-1 (PD-1), CD274 (PD-L1), BTLA, TIGIT, CTLA4, PDCD1LG2, and LAG3, among others, in the low-risk subsets compared with the high-risk subsets (Figure 8C). Figure 8B shows that, compared with the expression of m6A-related mRNAs between the low- and high-risk groups, the expression of RBM15, YTHDC2, WTAP, METTL14, YTHDC1, and METTL3 was differentiated.

### Correlation Between Risk Grouping and PAM50 Molecular Subtypes

**FIGURE 9:**
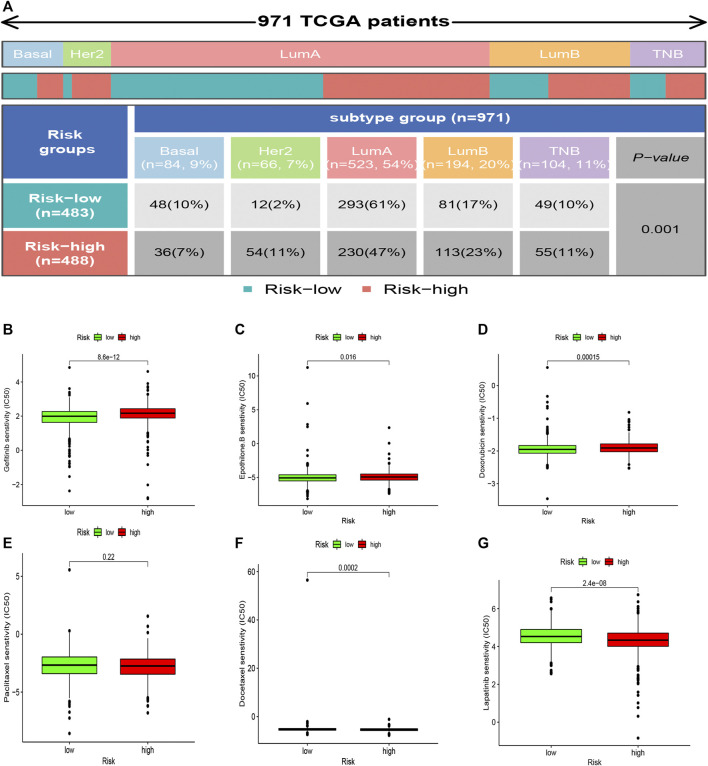
Heatmap and table showing the distribution of the BRCA PAM50 molecular subtypes (basal, luminal A, luminal B, HER2^+^, and TNBC) in the risk subgroups **(A)**. Relationships between the risk score and chemotherapeutic sensitivity **(B–G)**.

The high-risk subsets and low-risk subsets were split into PAM50 molecular subtypes (Parker et al., 2009), respectively, including basal, Her2+, luminal A, luminal B, and triple-negative breast cancer (TNBC), as shown in Figure 9A. In summary, the proportion of TNBC samples was almost equally distributed between the two groups, whereas there were more HER2+ samples and more luminal A samples in the high-risk subgroup (*p* < 0.001, χ2 test).

### Chemotherapy Sensitivity Related to the Risk Score

The correlation between the sensitivity to chemotherapeutic drugs and this prognostic model was explored. The IC50 values of usual chemotherapeutic drugs were predicted and compared between the low- and high-risk groups. Patients in the low-risk group were more responsive to gefitinib, epothilone B, and doxorubicin, whereas patients in the high-risk set were more responsive to docetaxel and Lapatinib. However, no statistical significance was observed regarding the differences in the response to paclitaxel (Figures 9B–G).

## Discussion

In recent years, researchers have dedicated increased efforts toward proving that the amino acid metabolism (AAM) is dramatically associated with BRCA development (Cha et al., 2018; Deyu Zhang et al., 2021; Morotti et al., 2021). Glutamine, serine, glycine, etc. are critical nutrients for tumor growth and maintenance. Similarly, amino acids are vital nutrients for immune cells. For example, Glutaminolysis is a major energy-producing process for proliferating cells, including activated T cells (Newsholme et al., 1999), by supplying a-ketoglutarate (aKG) to the TCA cycle, *via* glutamate. Glutamine is used to promote LPS induction of IL-1 production by macrophages (Wallace and Keast, 1992). Activated NK cells use some glutamine to replenish TCA cycle intermediates and increase oxidative phosphorylation (Lam et al., 2016). One function of the reduced form of glutathione (GSH) in Treg cells is to restrict serine metabolism in order to maintain their suppressive function. Macrophages also utilize serine to generate glycine for GSH, needed for LPS-induced IL-1b mRNA expression (Rodriguez et al., 2019). Consequently, increasing attention has been paid to AAM in this context (Jones et al., 2018; Butler et al., 2021). As expected, recent studies have reported that the combination of a glutamine antagonist and anti-PD-1 therapy had a more obvious anti-tumor effect than did the anti-PD-1 therapy alone, and did not cause immune cell failure (Leone et al., 2019). Moreover, previous studies have produced prognostic models of genes related with amino acid metabolism in glioma and hepatoma *via* bioinformatics analysis (Liu et al., 2019; Zhao et al., 2021). In recent years, lncRNAs have been proved that they have a great impact on the regulation of metabolism (Guo et al., 2021; Tan et al., 2021). To date, however, AAM-related lncRNAs have not been used to predict OS in patients with BRCA. Whether amino acid metabolization-related lncRNAs participate in the immune regulation of BRCA remains ambiguous.

In this study, we explored for the first time the characteristics of AAM-related lncRNAs in BRCA and established a risk signature associated with OS. First, we screened AAM-associated DEGs between BRCA and normal breast tissues based on RNA-seq data. Furthermore, we established a robust and effective prognostic signature using univariate Cox, LASSO regression, and multivariate Cox analyses. Nine lncRNAs were included in this signature (LIPE-AS1, AC124067.4, LINC01655, AP005131.3, AC015802.3, USP30-AS1, SNHG26, AL589765.4). Moreover, 1,005 samples in total were stochastically split into training and validation cohorts at a 3:1 ratio, for building and validating the AAM-related lncRNA signature. The training set of 754 samples, the validation set of 251 samples, and the whole TCGA data set of 1,005 samples all showed the feasibility of this model.

Several recent studies have demonstrated that LIPE-AS1 is prominently expressed in BRCA and cervical squamous cell carcinoma and is associated with a higher survival rate (Xu et al., 2021; Wang et al., 2022). In turn, AC124067.4-hsa-miR-92b-3p (hsa-miR-589-5p)-PHYHIPL both decrease the MSI and TMB in COAD, thus reducing the risk of genome instability and alterations (Ren et al., 2021), whereas USP30-AS1 promotes mitochondrial quality control in glioblastoma cells (Wang et al., 2021), the USP30-AS1/miR-299-3p/PTP4A1 pathway aggravates the malignant progression of cervical cancer (Wang et al., 2021), and SNHG26 promotes the metastasis, growth, and cisplatin resistance of tongue squamous cell carcinoma through the PGK1/Akt/mTOR signaling pathway (Jiang et al., 2022). Nevertheless, no studies have investigated the prognostic value of LINC01655, AP005131.3, AC015802.3, and AL589765.4 in patients with BRCA or other malignancies. The current findings show for the first time that these four lncRNAs were associated with the prognosis of BRCA. The potential role of the four lncRNAs needs to be further explored.

To shed light on the immunologic nature of the risk subgroups, gene mutations were explored in the different risk subgroups. We found that the most frequent mutation type was missense mutation, followed by frameshift deletion and nonsense mutation, as reported previously (2012). The most significant difference in mutations between the groups was observed for TP53 mutations, which were more frequent in high-risk samples than in low-risk samples (77 vs. 64%). TP53 mutation is not only the single most significant genetic event in cancer, but also linked with poorer patient outcomes and more aggressive disease in many malignant tumors (Vousden and Prives, 2005), particularly BRCA (Olivier et al., 2006; Shahbandi et al., 2020). TP53 can affect the cancer cell cycle through the p53/TGF *β* signaling pathway. In a study of stage III breast cancer, patients with TP53 mutations were shown to have worse disease free survival (DFS) following treatment with paclitaxel (*p* = 0.007) (Chrisanthar et al., 2011). Olivier et al. noted in a study of 1794 breast cancer patients that those with tumors harboring TP53 mutations in exons 5–8 of the gene had a worse risk of dying of breast cancer within 10 years following surgery (*p* < 0.0001) (Olivier et al., 2006).

In addition, there was a higher rate of PIK3CA mutation in the high-risk subgroup compared with the low-risk subgroup, which could mean that tumor growth in high-risk BRCA cases is promoted through the PI3K–AKT signaling pathway (Xing et al., 2019). Hence, high-risk patients with high TP53 and PIK3CA mutations have a poorer prognosis than do low-risk patients with low TP53 and PIK3CA mutations, in accordance with our survival results. Next, we speculated that low-risk patients benefit more from the immune checkpoint inhibitor therapy, based on the results presented in Figure 8C, especially among patients with TNBC cancer in both groups (Lyons, 2019; Kwapisz, 2021).

Here, we concluded that the risk score had a slight positive relationship with the TMB (Figure 6D), which implies that the TMB can help explain why the risk score affects prognosis to a certain degree; however, other possible mechanisms may be involved in this relationship. Recent studies reported that TMB-high tumors not only did not exhibit a higher susceptibility to immune checkpoint blockade (ICB) vs. TMB-low tumors, but also exhibited a significantly lower susceptibility to ICB in BRCA, prostate cancer, etc. (McGrail et al., 2021). Interestingly, high expression levels of CTLA-4 and TIGIT were correlated with favorable prognosis in breast cancer (Fang et al., 2020). These are consistent with our results. Furthermore, we shed light on the tumor microenvironment and composition of immune cell infiltrates. The results of these analyses (Figures 7A,B) indicated that CD8^+^ T cells, plasma cells, naïve CD8^+^ T cells, M1 macrophages, memory B cells, and endothelial cells were more abundant in the low-risk subgroup, whereas M2 macrophages, M0 macrophages, and fibroblasts were more common in the high-risk subgroup. A substantial body of research has revealed that a high level of infiltration of T cells, especially cytotoxic CD8^+^ T cells, predicts a beneficial outcome (Bindea et al., 2013; Gentles et al., 2015; Fridman et al., 2017). Considering the fibroblasts in the high risk group were significantly higher than those in the low risk group (*p* < 0.001), we speculated that the high risk group were more abundant in cancer-associated fibroblast (CAF) compared to the low risk group. CAF is the main cell component of tumor microenvironment (TMB). Studies have shown that CAFs can assist the immune escape of breast cancer cells, promote proliferation, invasion and metastasis of breast cancer cells, inhibit immune response (Shimura et al., 2018; Gok Yavuz et al., 2019;Ivy X Chen et al., 2019) and inhibit T cell infiltration (Lei Li et al., 2021). This result is in accordance with our study.

In most malignancies, M2 macrophages, which are a significant subtype of macrophages, have been shown to correlate with increased tumor cell proliferation, development of an invasive phenotype, and chronic inflammation, and these cells have been correlated with a poor outcome in breast, gastric, ovarian, bladder, and prostate cancers (Josephs et al., 2015; Ruffell and Coussens, 2015; Fridman et al., 2017). Conversely, a high density of M1 macrophages seems to be correlated with acute inflammation and imply a favorable prognosis among patients with HCC, NSCLC, gastric, or ovarian cancers (Josephs et al., 2015; Ruffell and Coussens, 2015; Fridman et al., 2017). The results of our study support these conclusion.

Moreover, we found that the low-risk samples had a more robust ability for damage repair, whereas the high-risk samples had more immunosuppressive cells and signals and tumor and metastasis-related signals, which implies that the high-risk subgroup exhibited characteristics of immunosuppression and active tumor progression.

Furthermore, there were different proportions of the PAM50 subtypes in the two subsets (Figure 9A), as we found that the high-risk subgroup possessed more HER2+ samples, which are more invasive. In contrast, the low-risk subgroup possessed more luminal A samples, which are less invasive. In summary, we concluded that the low-risk subgroup was characterized by lower tumor aggressiveness and an active immune response, whereas the high-risk subgroup was characterized by higher tumor aggressiveness and an immune-suppressive response.

Finally, we learned that patients in the low-risk group were more responsive to treatment with gefitinib, epothilone B, and doxorubicin. In contrast, patients in the high-risk group were more responsive to treatment with docetaxel (Figures 9B–G). This phenomenon might provide valuable clinical treatment recommendations for high- and low-risk groups.

There are still some limitations to this study. First, the data was obtained only from a single TCGA dataset. The analysis of multiple datasets would have been more convincing. Second, the associations were analyzed solely by statistical analysis and were not validated experimentally. Lastly, when exploring the immune microenvironment, we did not illustrate the signalling pathways of the target genes at a deeper level. We should investigate the specific mechanisms of the AAM-related prognostic lncRNAs and immune cells in the future. There is still a long way to go to considerably optimise personalised immunotherapy management.

## Conclusion

In conclusion, we attempted to shed light on the importance of AAM-associated lncRNAs in BRCA. The prognostic model built here might be acknowledged as an indispensable reference for predicting the outcome of patients with BRCA and help identify their immune and molecular characteristics. However, further studies are needed to illustrate this point.

## Data Availability

The datasets presented in this study can be found in online repositories. The names of the repository/repositories and accession number(s) can be found in the article/Supplementary Material.

## Supplementary Material

Supplementary Figure S1 Validation of the risk score in the validation set and the complete TCGA data set. **(A)** K–M analysis in the validation set. **(B)** K–M analysis in the complete TCGA data set. **(C)** Time-dependent ROC analysis in the validation set. **(D)** Time-dependent ROC analysis in the complete TCGA data set.

Supplementary Figure S2 AAM-related lncRNA signature, 11-lncRNA signature (Shen et al., 2020), 11 (2)-lncRNA signature(Li et al., 2021b) , and eight-lncRNA signature(Zhu et al., 2021), respectively, based on TCGA. **(A–D)** Respective Kaplan–Meier curve results, **(E–H)** respective AUC for the prediction of the 1-, 3-, and 5-year survival rate in BRCA.

Supplementary Figure S3 Heatmap for immune responses based on CIBERSORT, ESTIMATE, MCPcounter, ssGSEA, and TIMER algorithms among high and low risk group.

## Abbreviations

AAM, Amino acid metabolism; BRCA, breast cancer; C-index, Harrell’s concordance index; DEGs, differentially expressed genes; GO, Gene Ontology; KEGG, Kyoto Encyclopedia of Genes and Genomes; OS, overall survival; ROC, received operating characteristic; ssGSEA, single-sample gene set enrichment analysis.
