# Supplementary figures and images for: Amino Acid Metabolism-Related lncRNA Signature Predicts the Prognosis of Breast Cancer
Source: Front Genet. 2022 May 13;13:880387. doi: 10.3389/fgene.2022.880387 (PMC9136175; doi:10.3389/fgene.2022.880387)

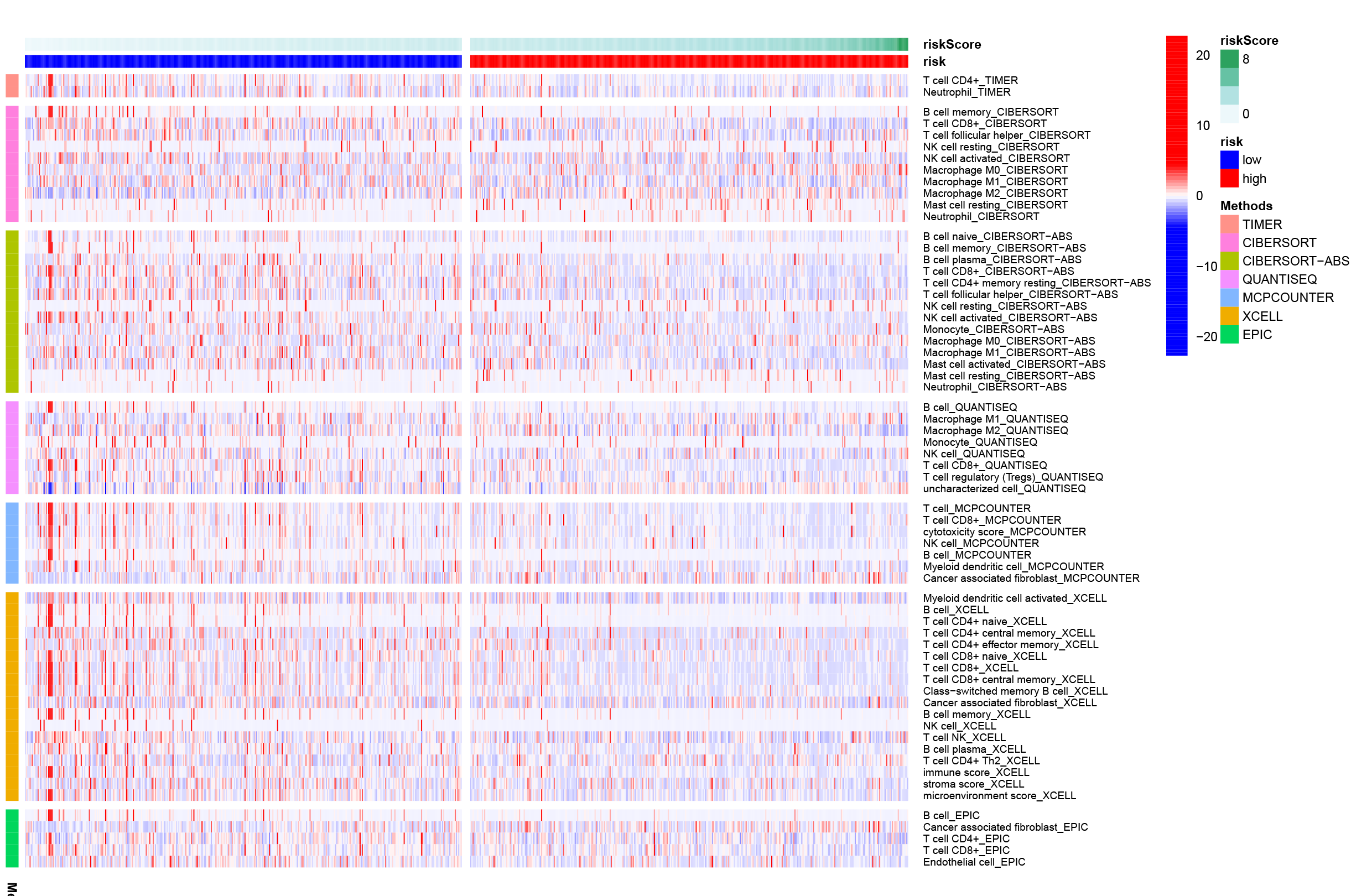

Supplement: Supplementary file 2 [file Image3.TIF]

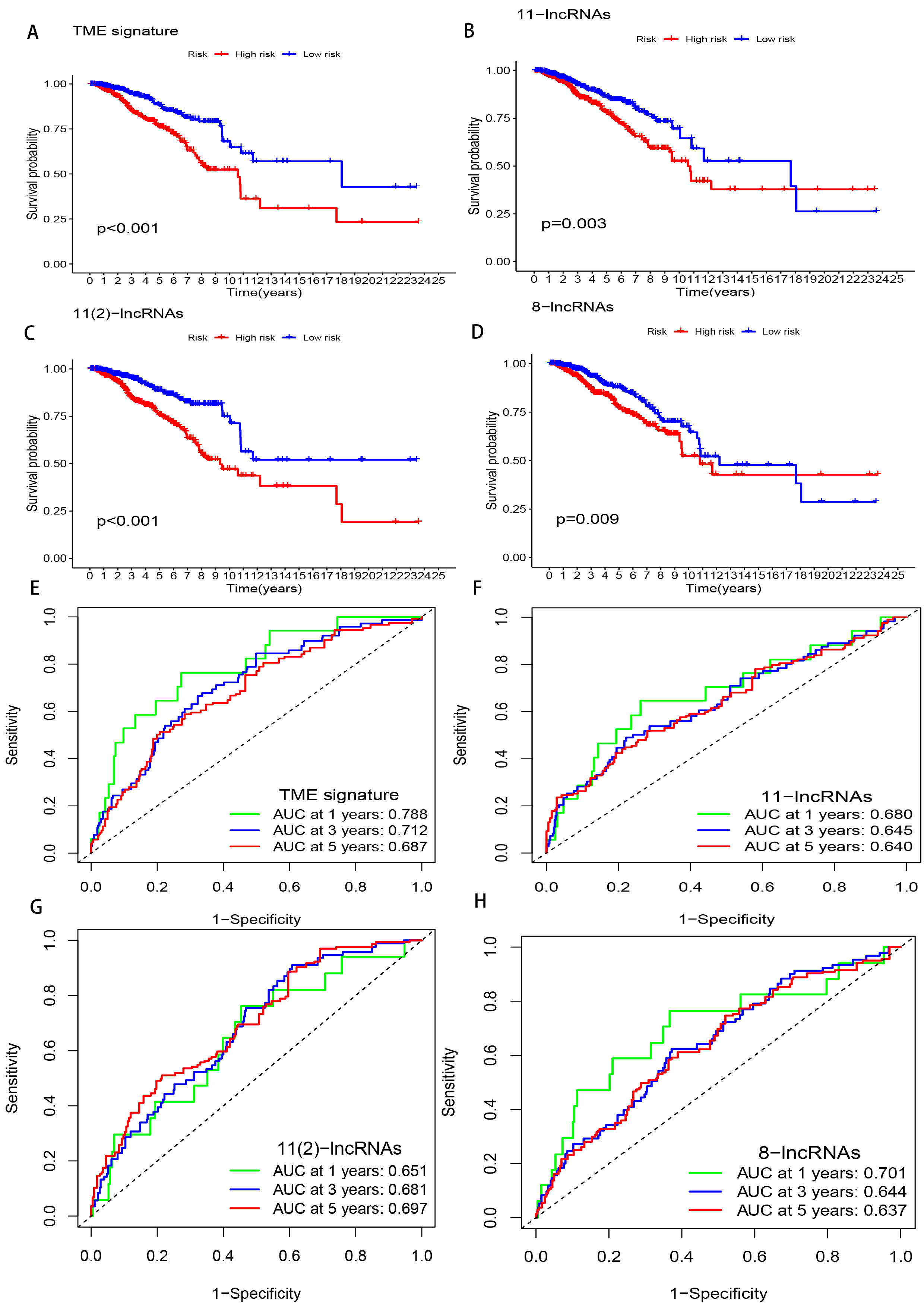

Supplement: Supplementary file 3 [file Image2.TIF]

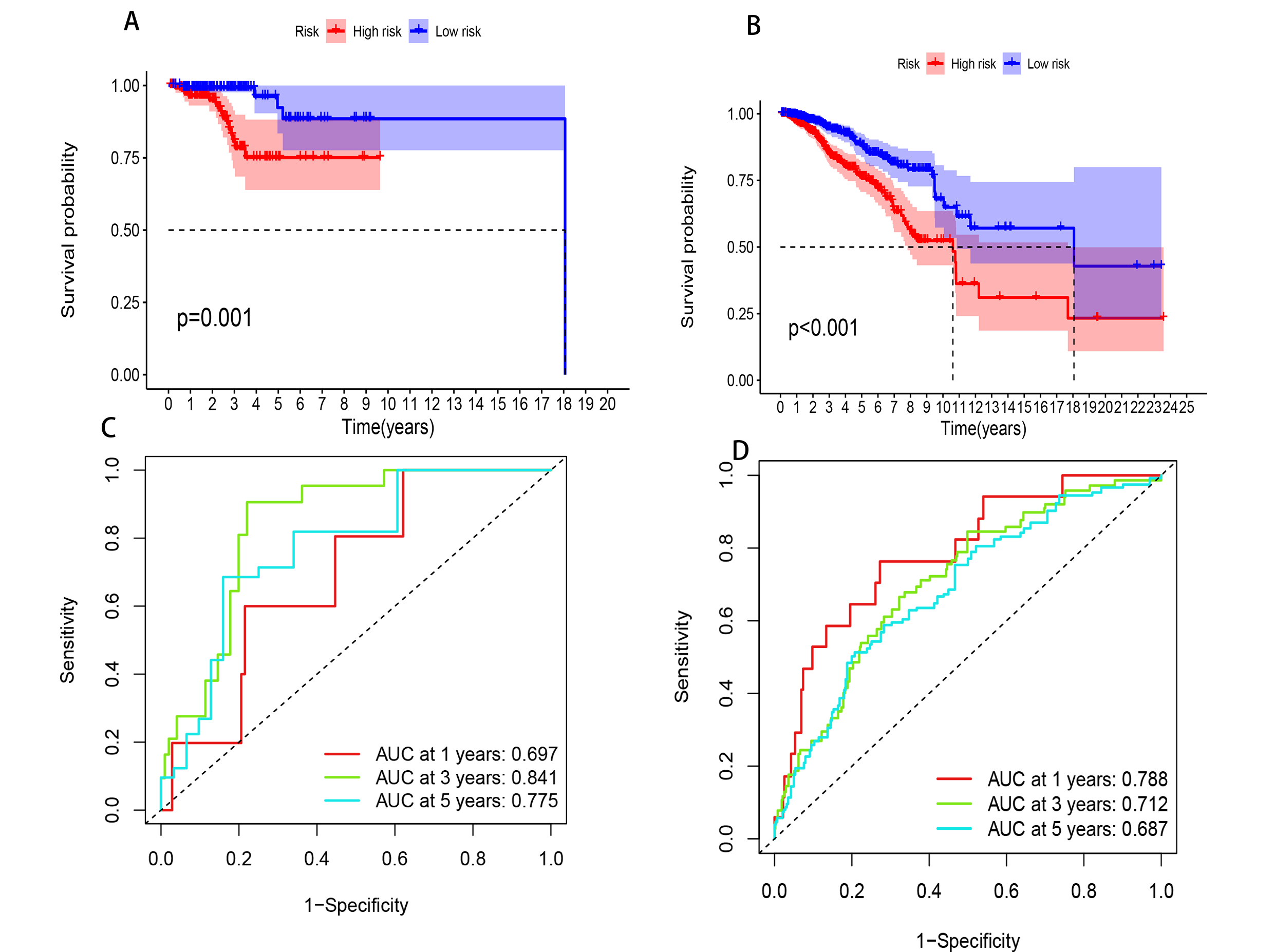

Supplement: Supplementary file 4 [file Image1.TIF]
